# Supplementary material for: Developing Endogenous Autophagy Reporters in Caenorhabditis elegans to Monitor Basal and Starvation-Induced Autophagy
Source: Int J Mol Sci. 2025 Oct 20;26(20):10178. doi: 10.3390/ijms262010178 (PMC12563416; doi:10.3390/ijms262010178)
Supplement: Supplementary file 1 [file ijms-26-10178-s001.zip › ijms-3877475-supplementary.pdf]

Developing Endogenous Autophagy Reporters in *Caenorhabditis elegans* to Monitor Basal and Starvation-Induced Autophagy

Kincső Bördén [1], Tibor Vellai [1,2,*] and Tímea Sigmond [1,*]

[1] Department of Genetics, Eötvös Loránd University (ELTE), Pázmány Péter sétány 1/C, 1117 Budapest, Hungary
[2] Hungarian Research Network-ELTE, Genetics Research Group, 1117 Budapest, Hungary
* Correspondence: vellai.tibor@ttk.elte.hu (T.V.); sigmond.timea@ttk.elte.hu (T.S.)

Supplementary Table S1. Statistical analyses of data.

Statistics tables

Genotype	Number of animals	Number of repeats	Mean Area% (a.u.)	±S.D. (%)	one-Vay ANOVA
**for Figure 2B″**					
**GFP signal**					
lgg-1(syb2459) [lgg-1p::gfp::mCherry::lgg-1]	21	3	0.08795	0.07719	-
**mCherry signal**					
lgg-1(syb2459) [lgg-1p::gfp::mCherry::lgg-1]	33	3	0.09671	0.09871	-

Genotype	Number of animals	Number of repeats	Mean Area% (a.u.)	±S.D. (%)	one-Vay ANOVA
**for Figure 3B″**					
**GFP signal**					
lgg-1(syb2459) [lgg-1p::gfp::mCherry::lgg-1]	27	3	0.02074	0.01549	
lgg-1(syb2459) [lgg-1p::gfp::mCherry::lgg-1];epg-5(tm3425) II.	27	3	0.03289	0.0147	p=0.0047
**mCherry signal**					
lgg-1(syb2459) [lgg-1p::gfp::mCherry::lgg-1]	30	3	0.2004	0.05295	
lgg-1(syb2459) [lgg-1p::gfp::mCherry::lgg-1];epg-5(tm3425) II.	27	3	0.09068	0.02979	p<0.0001

Genotype	Number of animals	Number of repeats	Mean Area% (a.u.)	±S.D. (%)	one-Vay ANOVA
**for Figure 3C″**					
**GFP signal**					
lgg-1(syb2459) [lgg-1p::gfp::mCherry::lgg-1]	21	3	0.008762	0.006811055	
lgg-1(syb2459) [lgg-1p::gfp::mCherry::lgg-1];hlh-30(tm1978) IV.	33	3	0.004538	0.004538	n.s.
**mCherry signal**					

lgg-1(syb2459) [lgg-1p::gfp::mCherry::lgg-1]	21	3	0.09671	0.007410831	
lgg-1(syb2459) [lgg-1p::gfp::mCherry::lgg-1];hlh-30(tm1978) IV.	33	3	0.001551	0.001976959	<0.0001

**Genotype**	**Number of animals**	**Number of repeats**	**Mean Area% (a.u.)**	**±S.D. (%)**	**one-Vay ANOVA**
**for Figure 3D”**					
**GFP signal**					
lgg-1(syb2459) [lgg-1p::gfp::mCherry::lgg-1] EV	19	3	0.02721	0.06789	
lgg-1(syb2459) [lgg-1p::gfp::mCherry::lgg-1] let-363 RNAi	22	3	0.01186	0.02391	p=0.9818
**mCherry signal**					
lgg-1(syb2459) [lgg-1p::gfp::mCherry::lgg-1] EV	24	3	1.076	0.3413	
lgg-1(syb2459) [lgg-1p::gfp::mCherry::lgg-1] let-363 RNAi	24	3	1.875	0.4293	p<0.0001

Genotype	Number of animals	Number of repeats	Mean Area% (a.u.)	±S.D. (%)	one-Vay ANOVA
**for Figure 4A′**					
**GFP signal**					<-> Well fed
lgg-1(syb2459) [lgg-1p::gfp::mCherry::lgg-1] well fed	28	3	0.007036	0.01376	
lgg-1(syb2459) [lgg-1p::gfp::mCherry::lgg-1] starved for 1 day	34	3	0.0004706	0.001926	p=0.0014
lgg-1(syb2459) [lgg-1p::gfp::mCherry::lgg-1] starved for 3 days	39	3	0.002077	0.007158	p=0.0163
lgg-1(syb2459) [lgg-1p::gfp::mCherry::lgg-1] starved for 5 days	42	3	4.76E-05	0.0003086	p=0.0003
**mCherry signal**					<-> Well fed
lgg-1(syb2459) [lgg-1p::gfp::mCherry::lgg-1] well fed	31	3	0.5499	0.168	
lgg-1(syb2459) [lgg-1p::gfp::mCherry::lgg-1] starved for 1 day	35	3	1.4	0.2608	p<0.0001
lgg-1(syb2459) [lgg-1p::gfp::mCherry::lgg-1] starved for 3 days	39	3	1.427	0.2154	p<0.0001
lgg-1(syb2459) [lgg-1p::gfp::mCherry::lgg-1] starved for 5 days	42	3	1.179	0.2682	p<0.0001

Genotype	Number of animals	Number of repeats	Mean Area% (a.u.)	±S.D. (%)	one-Vay ANOVA

**for Figure 4B′**					
**GFP signal**					<-> Well fed
lgg-1(syb2459) [lgg-1p::gfp::mCherry::lgg-1];epg-5(tm3425) II. Well fed	33	3	0.4527	0.5135	
lgg-1(syb2459) [lgg-1p::gfp::mCherry::lgg-1];epg-5(tm3425) II. starved for 1 day	30	3	0.395	0.3845	p=0.8826
lgg-1(syb2459) [lgg-1p::gfp::mCherry::lgg-1];epg-5(tm3425) II. starved for 3 days	31	3	0.2865	0.3165	p=0.2049
lgg-1(syb2459) [lgg-1p::gfp::mCherry::lgg-1];epg-5(tm3425) II. starved for 5 days	33	3	0.2393	0.2673	p=0.0662
**mCherry signal**					<-> Well fed
lgg-1(syb2459) [lgg-1p::gfp::mCherry::lgg-1];epg-5(tm3425) II. Well fed	33	3	2.813	0.6725	
lgg-1(syb2459) [lgg-1p::gfp::mCherry::lgg-1];epg-5(tm3425) II. starved for 1 day	30	3	2.081	0.6236	p<0.0001
lgg-1(syb2459) [lgg-1p::gfp::mCherry::lgg-1];epg-5(tm3425) II. starved for 3 days	31	3	1.581	0.5899	p<0.0001
lgg-1(syb2459) [lgg-1p::gfp::mCherry::lgg-1];epg-5(tm3425) II. starved for 5 days	33	3	1.518	0.5021	p<0.0001

**Genotype**	**Number of animals**	**Number of repeats**	**Mean Area% (a.u.)**	**±S.D. (%)**	**one-Vay ANOVA**
**for Figure 4C′**					
**GFP signal**					<-> Well fed

lgg-1(syb2459) [lgg-1p::gfp::mCherry::lgg-1] Well fed	21	3	0.008762	0.006811	
lgg-1(syb2459) [lgg-1p::gfp::mCherry::lgg-1] starved for 1 day	31	3	0.001545	0.003687	$p<0.0001$
lgg-1(syb2459) [lgg-1p::gfp::mCherry::lgg-1] starved for 3 days	26	3	0.0002528	0.001175	$p<0.0001$
lgg-1(syb2459) [lgg-1p::gfp::mCherry::lgg-1] starved for 5 days	25	3	0.0004	0.001118	$p<0.0001$
**mCherry signal**					<-> Well fed
lgg-1(syb2459) [lgg-1p::gfp::mCherry::lgg-1] Well fed	44	3	0.1255	0.05455	
lgg-1(syb2459) [lgg-1p::gfp::mCherry::lgg-1] starved for 1 day	31	3	0.2832	0.0817	$p<0.0001$
lgg-1(syb2459) [lgg-1p::gfp::mCherry::lgg-1] starved for 3 days	29	3	0.3793	0.1477	$p<0.0001$
lgg-1(syb2459) [lgg-1p::gfp::mCherry::lgg-1] starved for 5 days	28	3	0.2605	0.08341	$p<0.0001$

**Genotype**	**Number of animals**	**Number of repeat**	**Mean Area% (a.u.)**	**±S.D. (%)**	**one-Vay ANOVA**
**for Figure 5A**					
**GFP signal**					
lgg-1(syb2459) [lgg-1p::gfp::mCherry::lgg-1] Well fed	29	3	0.1343	0.1885	
lgg-1(syb2459) [lgg-1p::gfp::mCherry::lgg-1] starved for 1 day	27	3	0.02207	0.02755	p>0.9999 (<-> Well fed)
lgg-1(syb2459) [lgg-1p::gfp::mCherry::lgg-1] starved for 5 days	22	3	0.01009	0.02358	p<0.0001 (<-> Well fed)
lgg-1(syb2459) [lgg-1p::gfp::mCherry::lgg-1] starved for 5 days. then fed for 1 hour	28	3	0.02254	0.03337	p>0.9999 (<-> 5 days starved)
lgg-1(syb2459) [lgg-1p::gfp::mCherry::lgg-1] starved for 5 days. then fed for 3 hours	30	3	0.003533	0.01125	p>0.9999 (<-> 5 days starved)
lgg-1(syb2459) [lgg-1p::gfp::mCherry::lgg-1] starved for 5 days, then fed for 5 hours	23	3	0.001565	0.00529	p>0.9999 (<-> 5 days starved)
**mCherry signal**					
lgg-1(syb2459) [lgg-1p::gfp::mCherry::lgg-1] Well fed	30	3	1.018	0.2942	

lgg-1(syb2459) [lgg-1p::gfp::mCherry::lgg-1] starved for 1 day	27	3	2.377	0.653	p<0.0001 (<-> Well fed)
lgg-1(syb2459) [lgg-1p::gfp::mCherry::lgg-1] starved for 5 days	22	3	2.45	0.621	p<0.0001 (<-> Well fed)
lgg-1(syb2459) [lgg-1p::gfp::mCherry::lgg-1] starved for 5 days. then fed for 1 hour	28	3	0.997	0.3282	p<0.0001 (<-> 5 days starved)
lgg-1(syb2459) [lgg-1p::gfp::mCherry::lgg-1] starved for 5 days. then fed for 3 hours	30	3	1.179	0.3725	p<0.0001 (<-> 5 days starved)
lgg-1(syb2459) [lgg-1p::gfp::mCherry::lgg-1] starved for 5 days. then fed for 5 hours	23	3	0.927	0.333	p<0.0001 (<-> 5 days starved)
**Genotype**	**Number of animals**	**Number of repeats**	**Mean Area% (a.u.)**	**±S.D. (%)**	**one-Vay ANOVA**
**for Figure 5B**					
**GFP signal**					
lgg-1(syb2459) [lgg-1p::gfp::mCherry::lgg-1] Well fed	14	1	0.005286	0.01318	
lgg-1(syb2459) [lgg-1p::gfp::mCherry::lgg-1] starved for 1 day	13	1	0.001231	0.001166	p>0.9999 (<-> Well fed)

lgg-1(syb2459) [lgg-1p::gfp::mCherry::lgg-1] starved for 1 day + Bafilomycin A1	13	1	0.08931	0.05794	p=0.8826 (<-> 1 day no BafA)
lgg-1(syb2459) [lgg-1p::gfp::mCherry::lgg-1] starved for 3 days	13	1	0.0009231	0.00132	p>0.9999 (<-> Well fed)
lgg-1(syb2459) [lgg-1p::gfp::mCherry::lgg-1] starved for 3 days + Bafilomycin A1	11	1	0.05109	0.05634	p=0.9992 (<-> 3 days no BafA)
lgg-1(syb2459) [lgg-1p::gfp::mCherry::lgg-1] starved for 5 days	11	1	0.0002727	0.0006467	p>0.9999 (<-> Well fed)
lgg-1(syb2459) [lgg-1p::gfp::mCherry::lgg-1] starved for 5 days + Bafilomycin A1	8	1	0.4171	0.2077	p<0.0001 (<-> 5 days no BafA)
**mCherry signal**					
lgg-1(syb2459) [lgg-1p::gfp::mCherry::lgg-1] Well fed	14	1	0.5696	0.134	
lgg-1(syb2459) [lgg-1p::gfp::mCherry::lgg-1] starved for 1 day	13	1	1.4	0.1575	p<0.0001 (<-> Well fed)
lgg-1(syb2459) [lgg-1p::gfp::mCherry::lgg-1] starved for 1 day + Bafilomycin A1	13	1	0.7805	0.3995	p<0.0001 (<-> 1 day no BafA)
lgg-1(syb2459) [lgg-1p::gfp::mCherry::lgg-1] starved for 3 days	13	1	1.412	0.1954	p<0.0001 (<-> Well fed)

lgg-1(syb2459) [lgg-1p::gfp::mCherry::lgg-1] starved for 3 days + Bafilomycin A1	11	1	0.6915	0.154	p<0.0001(<-> 3 days no BafA)
lgg-1(syb2459) [lgg-1p::gfp::mCherry::lgg-1] starved for 5 days	11	1	1.195	0.1661	p<0.0001 (<-> Well fed)
lgg-1(syb2459) [lgg-1p::gfp::mCherry::lgg-1] starved for 5 days + Bafilomycin A1	8	1	0.6196	0.1285	p<0.0001 (<-> 5 days no BafA)
**Genotype**	**Number of animals**	**Number of repeats**	**Mean Area% (a.u.)**	**±S.D. (%)**	**one-Vay ANOVA**
**for Figure 7A**					
**GFP signal**					<-> Well fed
atg-5(syb2476) [atg-5p::gfp::atg-5] Well fed	9	1	11.84	1.575	
atg-5(syb2476) [atg-5p::gfp::atg-5] starved for 1 day	8	1	8.168	1.066	p<0.0001
atg-5(syb2476) [atg-5p::gfp::atg-5] starved for 3 days	5	1	7.896	1.237	p<0.0001
atg-5(syb2476) [atg-5p::gfp::atg-5] starved for 5 days	7	1	7.422	1.776	p<0.0001
atg-5(syb2476) [atg-5p::gfp::atg-5] starved for 7 days	7	1	5.777	1.551	p<0.0001
atg-5(syb2476) [atg-5p::gfp::atg-5] starved for 9 days	7	1	4.858	0.8742	p<0.0001

atg-5(syb2476) [atg-5p::gfp::atg-5] starved for 11 days	10	1	4.099	0.8655	p<0.0001

Genotype	Number of animals	Number of repeats	Mean Area% (a.u.)	±S.D. (%)	one-Vay ANOVA
**for Figure 7B**					
**GFP signal**					
atg-5(syb2476) [atg-5p::gfp::atg-5] Well fed	15	1	18.67	1.892	
atg-5(syb2476) [atg-5p::gfp::atg-5] starved for 1 day	12	1	10.22	0.9012	<0.0001
atg-5(syb2476) [atg-5p::gfp::atg-5] starved for 2 days	14	1	8.373	0.4738	<0.0001
atg-5(syb2476) [atg-5p::gfp::atg-5] starved for 3 days	11	1	2.692	0.9724	<0.0001

Genotype	Number of animals	Number of repeats	Mean Area% (a.u.)	±S.D. (%)	one-Vay ANOVA
**for Figure 7C**					
**GFP signal**					

atg-5(syb2476) [atg-5p::gfp::atg-5]; glo-4(ok623) V. Well fed	14	1	7	2.22	
atg-5(syb2476) [atg-5p::gfp::atg-5]; glo-4(ok623) V. starved for 1 day	10	1	0	0	$p<0.0001$

Supplementary Figure S1.

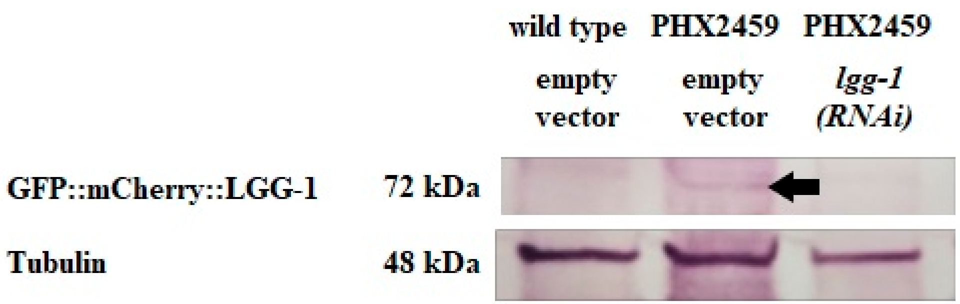

Suppl. Figure S1. Western blot analysis of GFP::mCherry::LGG-1 reveals the presence of the fusion protein in the transgenic strain (PHX2459). The arrows indicates the band stained by an mCherry antibody. Tubulin was used as an internal control.

Supplementary Method S1.

Sequences of the endogenous autophagy reporters.

Strain name and genotype: PHX2476 *atg-5(syb2476) [atg-5p::gfp::atg-5]*
Sequencing primers:
TBV01-seq-s: 5′-GAC TTG CTC ATT TTT GTG GA-3′
TBV01-seq-a: 5′-CAA GAA CTG TTT TTC CTG GT-3′
TBV01-mid-s: 5′-TTT CAG CCA ACA CTT GTC AC-3′
TBV01-mid-a: 5′-TTC CTG TAC ATA ACC TTC GG-3′
TBV01-syb2476

gacttgctcattttgtggaaaaacgttcaaattctgacatgaaagacagaaaaaaaaagttccaacttcgatttttaacgttttaatttttaattaaaaatgtttttttttgaaaaaaaaaattctaaaaaaattcggtttttttcaaaaatg
tggtaaaattcagatttcttatttaaaaaaaaacgtgaaaagttcgaaaattaagaaaaaatcgaatttcaggcaatttgtcggtttaccggaaattttcaagtccgaaaatttgccggtttgccggaaaaaaatagagaaaaaaatacagat
ttttcaaaaaaaaagtcccaaattcggtttttaatttttaaaaatcttttttttttaatttataaatcagagtgggcggcaatttgccgatttgccggaaataatcgtcgttccccgtttctataaatttctattttttttgcttacagATGAGTAA
AGGAGAAGAACTTTTCACTGGAGTTGTCCCAATTCTTGTTGAATTAGATGGTGATGTTAATGGGCACAAATTTTCTGTCAGTGGAGAGGGTGAAGGTG
ATGCAACATACGGAAAACTTACCCTTAAATTTATTTGCACTACTGGAAAACTACCTGTTCCATGGgtaagtttaaacatatatatactaactaaccctgattatttaaattttcagCC
AACACTTGTCACTACTTTCTgTTATGGTGTTCAATGCTTcTCgAGATACCCAGATCATATGAAACgGCATGACTTTTTCAAGAGTGCCATGCCCGAAGGT
TATGTACAGGAAAGAACTTATTTTTCAAAGATGACGGGAACTACAAGACACgtaagtttaaacagttcggtactaactaaccatacatatttaaattttcagGTGCTGAAGTCAAG
TTGAAGGTGTACCCTGTTATAGAATCGAGTTAAAAGGTATTGATTTTAAAGAAGATGGAAACATTCTTGGACACAAATTGGAATACAATATAACTCA
CACAATGTATACATCATGGCACAAACAAAAGAATGGAATCAAAGTTgtaagtttaaacatgattttactaactaactaatctgattaaattttcagAACTTCAAAATTAGACACAA
CATTGAAGATGGAAGCGTTCAACTACAGACATTATCAACAAATACTCCAATGGCGATGGCCCTGTCCTTTTACCAGACAACCATTACCTGTCCACAC
AATCTGCCCTTTCGAAAGATCCCAACGAAAAGAGAGACACATGGTCCTTCTTGAGTTTGTAACAGCTGCTGGGATTACACATGGCATGGATGAACTA
TACAAAGGCCACGGCACCGGCAGCACCGGCAGCGGCAGCAGCATGGACTAGAAGTATGCAGAAAA**GT**CTGGGAATCGCATGTTCCATGTC
AATTCACACTTCAAT**CT**TCTGGCGGAACTCACGGAGAACCTCTACCATTCTATACGATGCTCCCAAGATTCTCATATTTGGCTCTGGCAATACAGAAA
gttagattttctgagaaaaaaatcagcatttttccgataaatattgagatttttattaatttctcaaaaaattcaatttttaattaaaaacaaaagtttctgttatttttgggcaaaaaaaaaatcaaaaaccacaaaatcgtacctttttttgaata
aaaatgttgaaaaattggggaaaaaagaattaaagaatttttgggcaaaaaatcatgtaaattaattttttttttcgtaaaaatttgatctaaaaatagaaaaaaagcttgtttttttctaatttttcagtttttaccaggaaaaacagttcttg

Strain name and genotype: PHX2459 *lgg-1(syb2459) [lgg-1p::gfp::mCherry::lgg-1]*
Sequencing primers:
TS01-seq-s: 5′-CCC CGA CAA CCT CTA AAC-3′
TS01-seq-a: 5′-CAC ATT CGT CGG CGG ATA-3′
TS01-mid-s: 5′-TGC CCG AAG GTT ATG TAC AG-3′

TS01-syb2459

ccccgacaacctctaaacagctgcattgtacttgtcctgtttctctgcatctcaacaaaaccaggccagacaaacatagaaaatcaataggatgtctcctgcgtctacgcgctctcatatgaccgatgtgcgcgcacactccctctctcac
ttgcacacgggccattcttgctcatgctctttttgccttactagtactcgtcgtcaattttatatagatttcgcaatgtgttatcgaatcggtcaatctctcaaaacccatgtgatgtcaccttcattcactctcacttgttacacctttcttctctcatggtt
cttttatgatttttctgtgatttaaagtatttttattccagtaaccttctcttcacactaaccttcgaatcaaaATGAGTAAAGGAGAAGAACTTTTCACTGGAGTTGTCCCAATTCTTGTTGAATTAG
ATGGTGATGTTAATGGGCACAAATTTTCTGTCAGTGGAGAGGGTGAAGGTGATGCAACATACGGAAAACTTACCCTTAAATTTATTTGCACTACTGG
AAAACTACCTGTTCCATGGgtaagtttaaacatatatatactaactaaccctgattatttaaattttcagCCAACACTTGTCACTACTTTCTgTTATGGTGTTCAATGCTTcTCgAGATA
CCCAGATCATATGAAACgGCATGACTTTTTCAAGAGTGCCATGCCCGAAGGTTATGTACAGGAAAGAACTATATTTTTCAAAGATGACGGGAACTAC
AAGACACgtaagtttaaacagttcggtactaactaaccatacatatttaaattttcagGTGCTGAAGTCAAGTTTGAAGGTGATACCCTTGTTAATAGAATCGAGTTAAAAGGTATT
GATTTTAAAGAAGATGGAAACATTCTTGGACACAAATTGGAATACAACTATAACTCACACAATGTATACATCATGGCAGACAAACAAAAGAATGGA
ATCAAAGTTgtaagtttaaacatgattttactaactaactaatctgatttaaattttcagAACTTCAAAATTAGACACAACATTGAAGATGGAAGCGTTCAACTAGCAGACCATTAT
CAACAAAATACTCCAATTGGCGATGGCCCTGTCCTTTTACCAGACAACCATTACCTGTCCACACAATCTGCCCTTTCGAAAGATCCCAACGAAAAGA
GAGACCACATGGTCCTTCTTGAGTTTGTAACAGCTGCTGGGATTACACATGGCATGGATGAACTATACAAACAACAACAACAACAATCTCAACAAC
AACAACAATCTGTCTCAAAGGGT**GAG**GAAGATAACATGGCAATTATTAAAGAGTTTATGCGTTTCAAGGTGCATATGGAGGGATCTGTCAATGGGC
ATGAGTTTGAAATTGAAGGTGAAGGAGAAGGCCGACCATATGAGGGAACACAAACCGCAAAACTAAAGgtaagtttaaacatatatatactaactaaccctgattatttaaatt
ttcagGTAACTAAAGGCGGACCATTACCATTCGCCTGGGACATCCTCTCTCCACAGTTCATGTATGGAAGTAAAGCTTATGTTAAACATCCGGCAGATA
TACCAGATTATTTGAAACTTTCATTCCCGGAGGGTTTTAAGTGGGAACGCGTAATGAATTTTGAAGACGGAGGAGTTGTTACAGTGACGCAAGACTC
AAGgtaagtttaaacagttcggtactaactaaccatacatatttaaattttcagCCTCCAAGATGGAGAATTTATTTATAAAGTCAAACTTCGAGGAACGAATTTCCCCTCGGATGG
ACCTGTTATGCAGAAGAAGACTATGGGATGGGAAGCTTCAAGTGAAAGAATGTACCCTGAAGACGGTGCTCTTAAGGGAGAGATTAAACAACGTCT
TAAATTGAAAGATGGAGGACATTACGATGCTGAGgtaagtttaaacatgattttactaactaactaatctgatttaaattttcagGTGAAGACAACTTACAAAGCCAAAAAACCAG
TTCAGCTGCCAGGAGCGTACAATGTTAATATTAAACTGGATATCACCTCCCACAACGAGGATTACACTATCGTTGAGCAATATGAAAGAGCTGAAGG
GCGGCACTCGACAGGTGGCATGGATGAATTGTATAAGAAGTGG**GCG**TACAAGGAGGAGAACAACTTTGAGAAGCGTCGTGCCGAAGGAGACAAG
ATCCGCAGAAAGTACCCAGACCGTATTCCAGTGATTGTTGAGAAAGCACCAAAGTCAAAGCTCCATGACTTGGATAAGAAGAAGTACTTGGTCCCA
TCCGATCTTACTGTTGGACAGTTCTACTTCCTCATCAGAAAACGCATCCAACTTCGTCCAGAAGATGCTCTGTTCTTCTTTGTCAACAATGTCATTCCA
CAAACCATGACCACAATGGGACAACTCTACCAGgtaactaaccagtcgtttattttcatttaattaaccctttttctttattacagGACCATCACGAGGAAGACTTGTTCCTTTACATCG
CCTACAGTGACGAAAGTGTGTATGGAGGAGAGGTCGAAAAGAAGGAATAAagtgtcatgtattatccgccgacgaatgtg

Supplementary Method S2.

qPCR data

Gravid adult wild-type (N2) or PHX2459 l*gg-1(syb2459)* animals were allowed to lay eggs for 4-8 hours to obtain a synchronized population on NGM plates seeded with OP50 and maintained at 20°C until the animals reached the L4 stage. Starved animals were incubated in M9 for 24 hours at 20°C. while control animals remained on OP50 plates. After incubation. animals were collected and washed with M9 buffer.

RNA was isolated using RNAzol® RT (RN 190) (Molecular Research Center. INC.; 5645 Montgomery Road. Cincinnati. OH 45212. USA) and purified from the aqueous phase after extraction using RNA Clean & ConcentratorTM-5 kit (R1013) (Zymo Research Co.; 17062 Murphy Ave.. Irvine. CA 92614. USA). cDNA was synthesized using the RevertAid First Strand cDNA Synthesis Kit (K1622) (Thermo Fisher Scientific Inc.; 81 Wyman St. Waltham. MA 02451. USA).

Quantitative real-time PCR was performed using the Roche LightCycler® 96 System (F. Hoffmann-La Roche AG. Grenzacherstrasse 124. 4070 Basel. Switzerland) using the following primers: *lgg-1* Forward 5′-ACC CAG ACC GTA TTC CAG TG-3′. Reverse 5′-ACG AAG TTG GAT GCG TTT TC-3′. *cdc-42* Forward 5′-CTG CTG GAC AGG AAG ATT ACG-3′. Reverse 5′-CTC GGA CAT TCT CGA ATG AAG-3′. *gfp* Forward 5′-TCT GTC AGT GGA GAG GGT GAA-3′. Reverse 5′-GAC AAG TGT TGG CCA TGG AAC-3′.
